# The long noncoding RNA LINC01140/miR-140-5p/FGF9 axis modulates bladder cancer cell aggressiveness and macrophage M2 polarization

**DOI:** 10.18632/aging.202147

**Published:** 2020-11-21

**Authors:** Shuiqing Wu, Ran Xu, Xuan Zhu, Haiqing He, Jinhua Zhang, Qi Zeng, Yinhuai Wang, Xiaokun Zhao

**Affiliations:** 1 Department of Urology, The Second Xiangya Hospital, Central South University, Changsha 410011, Hunan Province, People’s Republic of China

**Keywords:** MIBC (muscle invasive bladder cancer), macrophage M2 polarization, long non-coding RNA LINC01140, miR-140-5p, FGF9

## Abstract

MIBC (muscle invasive bladder cancer) only accounts for only a minority of bladder cancers, however, the disease-specific and overall survival rates of patients with MIBC are low. Macrophage M2 polarization has been reported to be associated with poorer prognosis in bladder cancer. Through cancer bioinformatics and experimental analyses, FGF9 was found to be upregulated in MIBC tissues. FGF9 knockdown in T24 cells strongly suppressed the viability, migratory capacity, and invasive capacity of cells; culture with medium from FGF9 knockdown T24 cells (si-FGF9-CM) significantly inhibited macrophage M2 polarization, while promoting M1 polarization. The long noncoding RNA (lncRNA) LINC01140 was positively correlated with FGF9 and was significantly upregulated in MIBC tissues. LINC01140 knockdown inhibited the viability, migratory capacity and invasive capacity of T24 cells; culture in si-LINC01140-CM also inhibited macrophage M2 polarization, while promoting M1 polarization. LINC01140 targeted miR-140-5p, while miR-140-5p targeted FGF9 to form a lncRNA-miRNA-mRNA axis. The effects of miR-140-5p inhibition on bladder cancer aggressiveness and macrophage M2 polarization were opposite to those of LINC01140 or FGF9 knockdown; additionally, miR-140-5p inhibition partially reversed the effects of LINC01140 knockdown on FGF9 protein levels, bladder cancer phenotype, and macrophage M2 polarization. In conclusion, LINC01140, miR-140-5p, and FGF9 form a lncRNA-miRNA-mRNA axis that modulates the bladder cancer phenotype, affects macrophage M2 polarization through the tumor microenvironment, and in turn affects bladder cancer cell aggressiveness.

## INTRODUCTION

Bladder cancer is one of the most serious malignancies in the world [1]. At the first diagnosis, nearly three-quarters of bladder cancer patients are diagnosed with NMIBC (non-muscle invasive bladder cancer), while approximately a quarter of the patients have MIBC (muscle invasive bladder cancer) [2, 3]. Although MIBC only accounts for the minority of bladder cancers, disease-specific and overall survival rates are generally recognized to be lower in patients with MIBC [4, 5], with an overall five-year survival rate of 30–50% [3]. Maximal transurethral resection with chemoradiotherapy is the most common therapeutic strategy for bladder cancer [6]. However, MIBC is considered a highly lethal disease and it requires neoadjuvant treatment; therefore, comparing the differences between MIBC and NMIBC might provide some strategies for developing improved neoadjuvant treatment.

The tumor microenvironment (TME) is the environment around a tumor, and consists of tumor cells, stroma and infiltrating immune cells. Immune cells within the tumor microenvironment could play a role in inhibiting or promoting cancers [7]. Both CD8+ T cells and NKs (natural killer cells) can mediate anticancer effects, while tumor-related macrophages and regulatory T cells act as tumor promoters [8, 9]. Macrophages are critical players in the innate and adaptive immune systems. The latest classification of macrophage functional types comprises classically activated (M1) macrophages with pro-inflammatory activity and alternatively activated (M2) macrophages, with anti-inflammatory functions [10]. There is growing evidence that M2 macrophages can accelerate tumor progression and metastasis [8, 11, 12]. In bladder cancer, M2 or alternatively polarized macrophages are associated with poorer prognosis [13]. Thus, it is speculated that M2 macrophages can possibly serve as immunotherapeutic targets for the treatment of MIBC [12]. The accelerating effect of M2 macrophages on tumor progression and metastasis are mediated through complex cross-talk mechanisms by which cancer cells act on tumor-associated macrophages, and vice versa. However, the precise mechanisms by which bladder cancer cells reeducate resident macrophages to elicit this polarization program remain elusive.

Over the past few decades, high-throughput biometric technology has been applied in cancer research to determine the critical biomarkers capable of facilitating discovery and translation [14, 15]. Currently, we can perform advanced bioinformatic analysis to address aberrant gene expression and critical signaling pathways, thus providing multiple clinical assessments for the research of bladder cancer [16]. Fibroblast growth factor 9 (FGF9) has been reported to enhance M2 macrophage polarization in the infarcted diabetic heart [17] while M2 macrophages are the main type of bladder cancer-related macrophages and can accelerate bladder cancer progression [18, 19]. In cancers, FGF9 overexpression accelerates lung adenocarcinoma [20]. Notably, GSE77952 analysis identified FGF9 as a significantly upregulated gene in MIBC, in comparison with NMIBC; thus, investigating the effect of FGF9 on macrophage M2 polarization in bladder cancer and the potential mechanism could be suggested as a new strategy to treat MIBC.

As high throughput sequencing technologies evolve, it has been found that less than 2% of the transcripts encode proteins, whereas the rest are transcribed as ncRNAs (noncoding RNAs), which include miRNAs and lncRNAs (long noncoding RNAs) [21]. LncRNAs contribute to chromatin remodeling, RNA decay, epigenetic modulation, chromatin modification and many other cell functions [22, 23], by transcriptionally and posttranscriptionally affecting gene expression. In doing so, lncRNAs modulate tumor formation, invasion and metastasis [24, 25]. Notably, several lncRNAs have been reported to regulate macrophage M2 polarization in cancers. LncRNA GAS5 suppresses transcription of TRF4, a critical factor for modulating the polarization of M2 macrophages, thus inhibiting M2 polarization in demyelinating diseases [26]. LncRNA KCNQ1OT1 can suppress miR-21a-5p to induce the polarization of macrophages, thus improving particle-induced osteolysis [27]. In endometrial cancer, lncRNA NIFK-AS1 inhibits macrophage M2 polarization by targeting miR-146a [28]. Since many lncRNAs are deregulated in bladder cancer, we hypothesized that lncRNAs might also modulate macrophage M2 polarization, therefore affecting bladder cancer cell aggressiveness. In addition, the mechanism might be related to miRNAs.

Herein, we first examined FGF9 expression and protein levels in MIBC and NMIBC tissue samples; in addition, the protein content and distribution of the M2 marker CD163 were examined in the same samples using immunofluorescence (IF) staining. FGF9 was knocked down and the specific effects of FGF9 knockdown on bladder cancer cell aggressiveness and the M2 polarization of macrophages cultured in FGF9 knockdown cancer cell culture medium were determined. Next, differentially expressed lncRNAs related to FGF9 were analyzed, and LINC01140 was selected; the roles of LINC01140 in the aggressiveness of bladder cancer cells and the M2 polarization of macrophages cultured in cancer cell culture medium were determined. Finally, we analyzed miRNAs that might simultaneously target LINC01140 and FGF9 and selected miR-140-5p; the dynamic effects of LINC01140 and miR-140-5p on FGF9 expression, bladder cancer cell aggressiveness and the M2 polarization of macrophages cultured in cancer cell culture medium were determined. In summary, we identified a lncRNA-miRNA-mRNA axis modulating the macrophage M2 polarization, therefore affecting bladder cancer cell aggressiveness.

## RESULTS

### Expression of FGF9 in MIBC (muscular invasive bladder cancer) and NMIBC (non-muscular invasive bladder cancer) tissues

**Figure 1:**
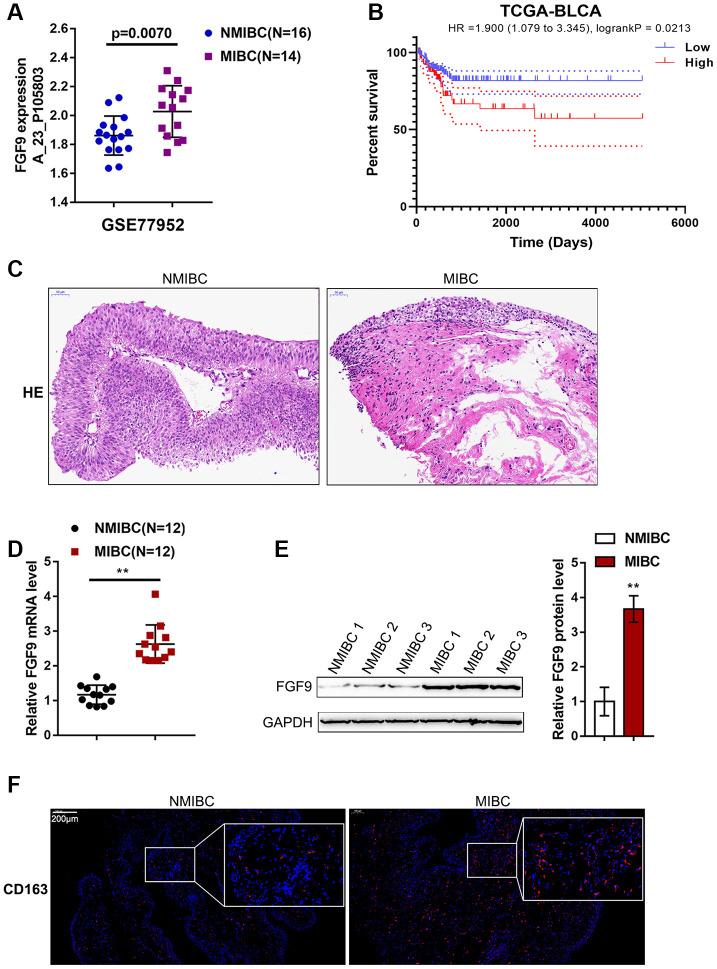
**Expression of FGF9 in muscular invasive bladder cancer (MIBC) and non-muscular invasive bladder cancer (NMIBC) tissues.** (**A**) The expression of FGF9 in MIBC and NMIBC tissues based on the data from GSE77952. *P*=0.007, student’s T test. (**B**) The overall survival of patients with bladder cancer was analyzed by grouping the samples based on FGF9 expression using log-rank test. Data are based on TCGA database. (**C**) The pathological changes in MIBC and NMIBC tissues was shown by H&E staining. (**D**) The expression of FGF9 in tissues of 12 cases of MIBC and 12 cases of NMIBC was determined using real-time PCR. *P*<0.01, paired student’s T test. (**E**) The protein levels of FGF9 in tissues of 12 cases of MIBC and 12 cases of NMIBC were determined using immunoblotting. *P*<0.01, paired student’s T test. (**F**) The protein distribution of CD163 (M2 macrophage marker) was determined in MIBC and NMIBC tissues using immunofluorescence (IF) staining.

To identify factors involved in MIBC pathogenesis, we analyzed an online microarray expression profile (GSE77952) reporting the differentially-expressed genes in tissues between 14 cases of MIBC and 16 cases of NMIBC; in the MIBC tissues, FGF9 expression was obviously upregulated (p=0.0070, Figure 1A). According to TCGA data, higher expression of FGF9 was correlated with poorer overall survival in bladder carcinoma patients (Hazard ratio = 1.900 (95% CI = 1.079-3.345), logrank P = 0.0213, Figure 1B). Thus, FGF9 was selected for further experiments.

Next, we collected 12 MIBC and NMIBC tissues, and the H&E staining results are shown in Figure 1C. Then, the expression of FGF9 in the tissues of 12 cases of MIBC and 12 cases of NMIBC was monitored using real-time PCR. Consistent with online data, the expression of FGF9 was dramatically upregulated in MIBC tissue samples compared with NMIBC tissue samples (Figure 1D). Immunoblotting analysis showed that FGF9 protein contents were increased within MIBC tissue samples compared with NMIBC tissue samples (Figure 1E). The protein content and distribution of CD163 (M2 macrophage marker) were determined in MIBC and NMIBC tissues using immunofluorescence (IF) staining; notably, the results showed that CD163-positive cells were more frequently observed in MIBC samples (Figure 1F). These data suggest that FGF9 is overexpressed in bladder cancer, especially in MIBC. In addition, M2 macrophages account for a greater proportion of macrophages in MIBC. Thus, we speculate that FGF9 might promote bladder cancer development, possibly by promoting macrophage M2 polarization.

### Effects of FGF9 on bladder cancer cell aggressiveness and macrophage M2 polarization

**Figure 2:**
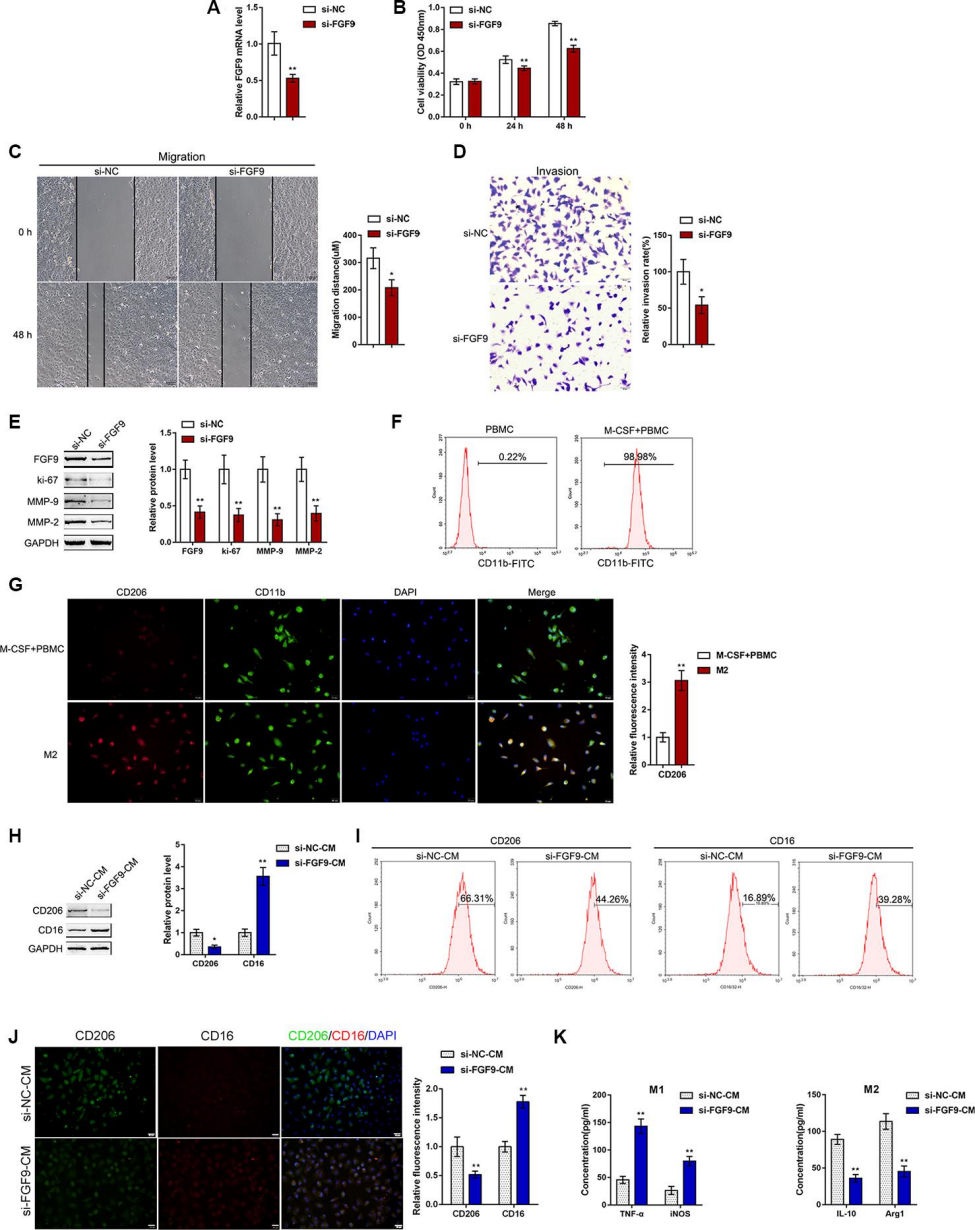
**Effects of FGF9 on bladder cancer cell aggressiveness and macrophage M2 polarization.** (**A**) FGF9 knockdown was generated in the T24 bladder cancer cell line by transfection of si-FGF9. The transfection efficiency was validated by real-time PCR. *P*<0.01, student’s T test. Next, T24 cells were transfected with si-FGF9 and examined for (**B**) cell viability by MTT assay; **P<0.01, one-way ANOVA test. (**C**) migration capacity by wound healing assay; *P*<0.05, student’s T test. (**D**) the invasive capacity by Transwell assay, *P*<0.05, student’s T test.; and (**E**) protein levels of FGF9, ki-67, MMP-2, and MMP-9 by immunoblotting. **P<0.01, student’s T test. (**F**) Monocytes were isolated from peripheral blood (PBMCs) and treated with 50 ng/ml M-CSF to stimulate the monocyte differentiation into macrophages (M0). The M0 macrophages were identified as CD11b positive by flow cytometry. (**G**) M0 macrophages were then stimulated with 20 ng/ml IL-4 (eBioscience) for two days to induce M2 polarization and authenticated using IF staining with anti-CD11b and anti-CD206 antibodies. The inflorescence intensity is shown in the right panel. *P*<0.01, student’s T test. (**H**–**K**) Next, T24 cells were transfected with si-FGF9 or si-NC (negative control) and the culture medium (shown in the figures as conditioned medium, si-NC-CM and si-FGF9-CM) was collected for macrophage incubation. M0 macrophages were divided into four groups: IL-4 (M2 polarization inducing) + si-NC-CM, IL-4 (M2 polarization inducing) + si-FGF9-CM, LPS + IFNγ (M1 polarization inducing) + si-NC-CM, and LPS + IFNγ (M1 polarization inducing) + si-FGF9-CM, and examined for (**H**) the protein levels of CD206 and CD16 by immunoblotting. *P*<0.05 or *P*<0.01, student’s T test.; (**I**) the percentage of CD206 and CD16 positive cells was determined by flow cytometry; (**J**) the inflorescence intensity of CD206 and CD16 was measured by IF staining. The inflorescence intensity is shown in the right panel. *P*<0.01, student’s T test. (**K**) The concentrations of IL-10, Arg1, iNOS, and TNF-α in the culture medium was determined by ELISA. **P<0.01, student’s T test.

To validate the hypothesis, we transfected si-FGF9 into T24 cells to generate FGF9 knockdown T24 bladder cancer cells, and performed real-time PCR (Figure 2A) and immunoblotting (Figure 2E) to verify the transfection efficiency. After knocking down FGF9 in T24 cells, cell viability, migration capacity, and invasive capacity were all significantly inhibited (Figure 2B-D). We further confirmed that the protein levels of the proliferation marker ki-67 and extracellular matrix markers MMP-2 and MMP-9 were also significantly decreased (Figure 2E). Thus, knocking down FGF9 significantly inhibits the aggressiveness of bladder carcinoma cells.

To validate the effects of FGF9 on macrophage M2 polarization in bladder cancer, we isolated monocytes from peripheral blood (PBMCs) and stimulated the monocytes with 50 ng/ml M-CSF for polarization induction. The macrophages were identified as CD11b positive by flow cytometry (Figure 2F). The polarized M2 macrophages were identified by IF for CD11b and CD206 expression. The results showed that the inflorescence intensity of CD206 expression was dramatically increased after M2 polarization (Figure 2G). Next, T24 cells were transfected with si-FGF9 or si-NC (negative control) and the culture medium (shown in the figures as conditioned medium si-NC-CM and si-FGF9-CM) was collected for macrophage incubation. M0 macrophages were divided into four groups: IL-4 (M2 polarization inducing) + si-NC-CM, IL-4 (M2 polarization inducing) + si-FGF9-CM, LPS + IFNγ (M1 polarization inducing) + si-NC-CM, and LPS + IFNγ (M1 polarization inducing) + si-FGF9-CM, and then the M1/M2 polarization markers were examined. Under M2 polarization conditions, culture with si-FGF9-CM significantly decreased the protein levels of M2 macrophage marker CD206 while increasing the M1 macrophage marker CD16 (Figure 2H). Moreover, the flow cytometry results and IF results further confirmed that si-FGF9-CM decreased the percentage of CD206 positive cells and CD206 fluorescence intensity while increasing the percentage of CD16 positive cells and CD16 fluorescence intensity (Figure 2I, 2J). ELISA also revealed that the concentrations of the M2 markers IL-10 and Arg1 were increased in si-FGF9-CM treated cells and that the concentrations of M1 markers iNOS and TNF-α were decreased in si-FGF9-CM treated cells (Figure 2K). These data indicate that FGF9 knockdown in bladder cancer cells could attenuate the macrophage M2 polarization in through the cancer microenvironment.

### Expression of the lncRNA LINC01140 in NMIBC and MIBC tissues

**Figure 3:**
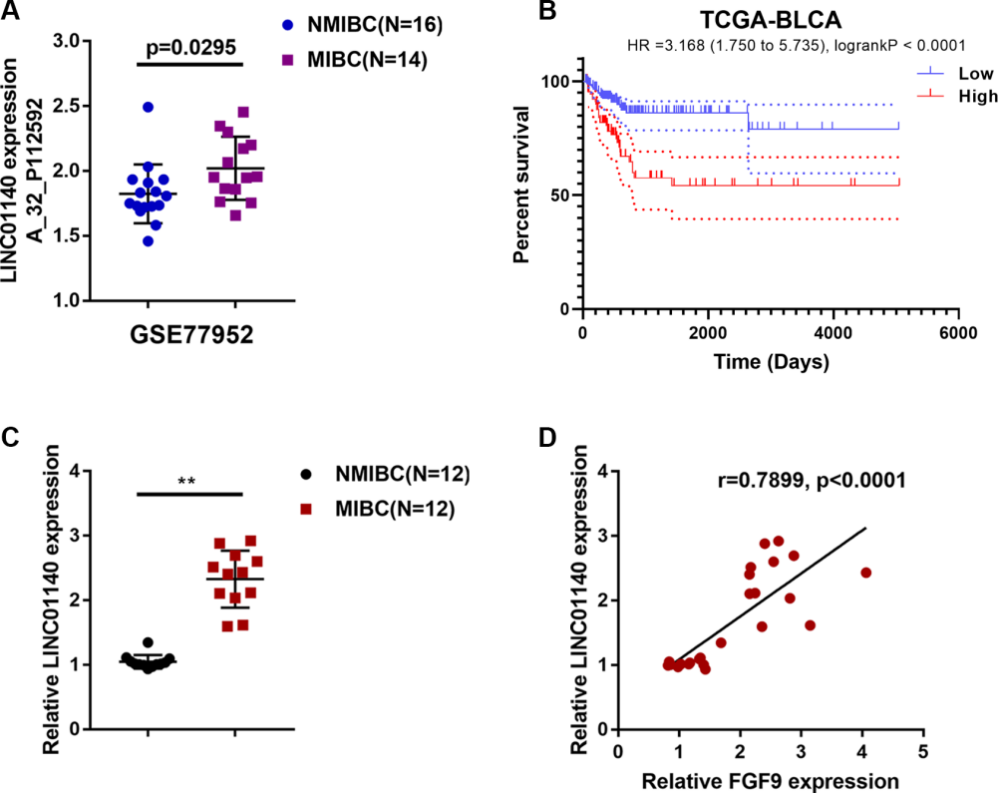
**Expression of lncRNA LINC01140 in NMIBC and MIBC tissues.** (**A**) The expression of lncRNA LINC01140 in MIBC and NMIBC tissues based on the data from GSE77952. *P*=0.0295, student’s T test. (**B**) The overall survival of patients with bladder cancer was analyzed by grouping the samples based on LINC01140 expression using log-rank test. Data are based on TCGA database. (**C**) The expression of LINC01140 in tissues of 12 cases of MIBC and 12 cases of NMIBC was determined using real-time PCR. *P*<0.01, paired student’s T test. (**D**) The correlation between LINC01140 and FGF9 expression in tissue samples was analyzed using Pearson’s correlation analysis. **P<0.01.

Considering that lncRNAs exert an essential effect on macrophage polarization and carcinogenesis [29–31], next, we analyzed GSE77952 to identify differentially- expressed lncRNAs that might be related to MIBC development. By analyzing the correlation between FGF9 and differentially- expressed lncRNAs, the study demonstrated that lncRNA LINC01140 expression had a significant correlation with FGF9 (data not shown) and was upregulated in MIBC tissues (p=0.0295, Figure 3A). In addition, based on TCGA data, higher expression of FGF9 was also associated with worse overall survival in bladder carcinoma patients (Hazard ratio = 3.168 (95% CI = 1.750-5.735), logrank P = 2.355e -05, Figure 3B).

Next, LINC01140 expression was determined in tissues from 12 cases of MIBC and 12 cases of NMIBC tissue using real-time PCR; similar to FGF9, the expression of LINC01140 was remarkably upregulated in MIBC tissue samples compared with NMIBC tissue samples (Figure 3C). In tissue samples, LINC01140 and FGF9 expression was positively correlated (Figure 3D).

### Effects of LINC01140 upon the aggressiveness of bladder carcinoma cells and the polarization of macrophage M2

**Figure 4:**
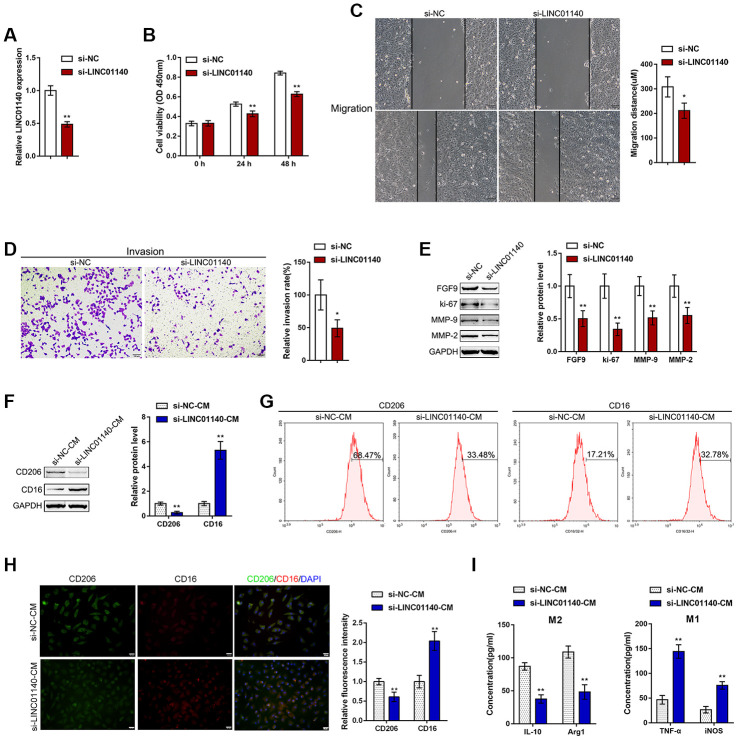
**Effects of LINC01140 on bladder cancer cell aggressiveness and macrophage M2 polarization.** (**A**) LINC01140 knockdown was generated in the T24 bladder cancer cell line by transfection with si-LINC01140. The transfection efficiency was validated by real-time PCR (*P*<0.01, student’s T test). Next, T24 cells were transfected with si-LINC01140 and examined for (**B**) cell viability by MTT assay, **P<0.01, one-way ANOVA test.; (**C**) migration capacity by wound healing assay, *P*<0.05, student’s T test; (**D**) invasive capacity by Transwell assay, *P*<0.05, student’s T test; and (**E**) protein levels of FGF9, ki-67, MMP-2, and MMP-9 by immunoblotting, *P*<0.01, student’s T test. T24 cells were transfected with si-LINC01140 or si-NC (negative control) and the culture medium (shown in the figures as conditioned medium, si-NC-CM and si-LINC01140-CM) was collected for macrophage incubation. Monocytes were treated with 50 ng/ml M-CSF to stimulate monocyte differentiation into M0 macrophages. M0 macrophages were divided into four groups: IL-4 (M2 polarization inducing) + si-NC-CM, IL-4 (M2 polarization inducing) + si-LINC01140-CM, LPS + IFNγ (M1 polarization inducing) + si-NC-CM, and LPS + IFNγ (M1 polarization inducing) + si-LINC01140-CM, and examined for (**F**) the protein levels of CD206 and CD16 by immunoblotting, *P*<0.01, student’s T test; (**G**) the percentage of CD206 and CD16-positive cells was determined by flow cytometry; (**H**) the inflorescence intensity of CD206 and CD16 were measured by IF staining, The inflorescence intensity is shown in the right panel. *P*<0.01, student’s T test. (**I**) The concentrations of IL-10, Arg1, iNOS, and TNF-α in the macrophage culture medium was determined by ELISA. **P<0.01, student’s T test.

To validate the specific effect of LINC01140 on the aggressiveness of bladder carcinoma cells and the development of MIBC, we transfected si-LINC01140 to generate LINC01140 knockdown in T24 cells, and performed real-time PCR to verify the transfection efficiency (Figure 4A). Similar to FGF9 knockdown, LINC01140 knockdown significantly inhibited T24 cell viability, migration and invasiveness (Figure 4B–4D). The protein levels of FGF9, ki-67, MMP-2, and MMP-9 were also obviously decreased by LINC01140 knockdown (Figure 4E).

Next, similarly, T24 cells were transfected with si-LINC01140 or si-NC (negative control) and the culture medium (shown in the figures as conditioned medium si-NC-CM and si-LINC01140-CM) was collected for macrophage incubation. M0 macrophages were divided into four groups: IL-4 (M2 polarization inducing) + si-NC-CM, IL-4 (M2 polarization inducing) + si-LINC01140-CM, LPS + IFNγ (M1 polarization inducing) + si-NC-CM, and LPS + IFNγ (M1 polarization inducing) + si-LINC01140-CM. M1/2 polarization markers were then examined. Under M2 polarization conditions, culture with si-LINC01140-CM significantly decreased the protein levels of the M2 marker CD206 while increasing the M1 marker CD16 in macrophages (Figure 4F). Moreover, the flow cytometry results and IF results further confirmed that si- LINC01140-CM decreased the percentage of CD206-positive cells and CD206 in fluorescence intensity while increased the percentage of CD16 positive rate and CD16 fluorescence intensity (Figure 4G and Figure 4H). Moreover, the concentrations of M2 markers IL-10 and Arg1 were significantly decreased, while the concentrations of the M1 markers iNOS and TNF-α were increased in si-LINC01140-CM-treated cells (Figure 4G). These data suggest that LINC01140 might also act as an oncogene in bladder cancer, possibly by affecting bladder cancer cell aggressiveness and macrophage M2 polarization.

### miR-140-5p directly targets LINC01140 and 3'UTR of FGF9

**Figure 5:**
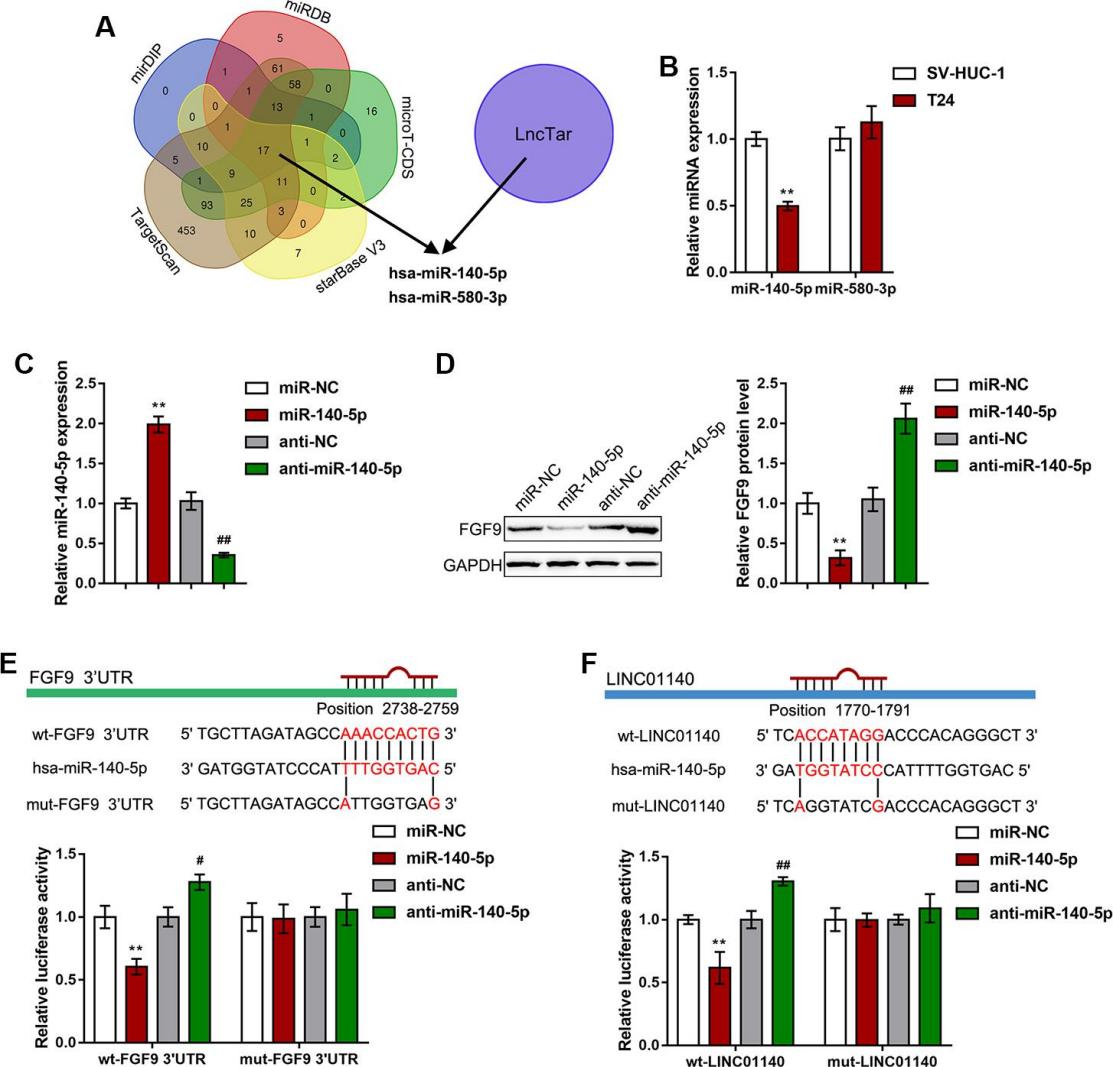
**miR-140-5p directly targets LINC01140 and the FGF9 3'UTR.** (**A**) miRNAs that might simultaneously target both LINC01140 and FGF9 3'UTR were predicted and selected by the online tools, mirDIP, TargetScan, starBase V3, microT-CDS, miRDB, and LncTar. Two miRNAs, miR-140-5p and miR-580-3p were selected. (**B**) The expression of miR-140-5p and miR-580-3p was determined in SV-HUC-1 and T24 cells by real-time PCR. *P*<0.01, student’s T test. (**C**) miR-140-5p was overexpressed or inhibited in T24 cells by the transfection of miR-140-5p or anti-miR-140-5p. The transfection efficiency was validated by real-time PCR. *P*<0.01, one way ANOVA test. (**D**) T24 cells were transfected with miR-140-5p or anti-miR-140-5p and examined for the protein levels of FGF9 by immunoblotting, *P*<0.01, one way ANOVA test. (**E**, **F**) Wild-type and mutant LINC01140 and FGF9 3'UTR luciferase reporter vectors were constructed as described in the Materials and methods section. These vectors were cotransfected with miR-140-5p or anti-miR-140-5p in 293T cells. The changes in luciferase activity were determined. *P*<0.01, one way ANOVA test. **P<0.01, #*P*<0.05, ##*P*<0.01.

Since lncRNAs can competitively target miRNAs via their MREs (miRNA response elements) to act as ceRNAs (competing endogenous RNAs), thus regulating the expression of downstream target RNAs [40], here, we hypothesized that miRNAs might be involved in the effect of LINC01140 and FGF9 on the aggressiveness of bladder carcinoma cells and macrophage M2 polarization. We used six online tools (mirDIP, TargetScan, starBase V3, microT-CDS, miRDB, and LncTar) to identify miRNAs that might simultaneously target both LINC01140 and the FGF9 3'UTR; we selected two miRNAs, miR-140-5p and miR-580-3p (Figure 5A). Next, we performed real-time PCR to examine miR-140-5p and miR-580-3p expression in both T24 cells and a normal human bladder epithelial cell line, SV-HUC-1; the expression of miR-140-5p was considerably downregulated in T24 cells while miR-580-3p expression showed no significant changes (Figure 5B). Thus, we selected miR-140-5p for further experiments.

To investigate the specific effect of miR-140-5p, we transfected miR-140-5p/ anti-miR-140-5p to overexpress or inhibit miR-140-5p in T24 cells, and performed real-time PCR to verify the transfection efficiency (Figure 5C). In T24 cells, miR-140-5p overexpression remarkably reduced FGF9 protein levels, while miR-140-5p inhibition increased FGF9 protein levels (Figure 5D). To further confirm the relevance of miR-140-5p to LINC01140 and FGF9, we measured the levels of miR-140-5p in response to LINC01140 and FGF9 knockdown. As shown in Supplementary Figure 2A, knockdown of LINC01140 increased the levels of miR-140-5p, while knockdown of FGF9 did not affect the miR-140-5p levels (Supplementary Figure 2B).

To validate the predicted binding of miR-140-5p and LINC01140 and FGF9 3'UTR, a luciferase reporter assay was performed. Based on the Materials and Methods section, we constructed two different types of LINC01140 and FGF9 3'UTR luciferase reporter vectors, wild-type and mutant. Second, we cotransfected these vectors with miR-140-5p/anti-miR-140-5p in 293T cells. Figure 5E, 5F shows that wild-type LINC01140 and FGF9 3'UTR vector luciferase activity was obviously inhibited by the overexpression of miR-140-5p but enhanced by the inhibition of miR-140-5p. Furthermore, mutating the putative miR-140-5p binding site eliminated the changes in luciferase activity. These data indicate that LINC01140 and FGF9 3'UTR are the direct downstream targets of miR-140-5p.

### Dynamic effects of LINC01140 and miR-140-5p on the aggressiveness of bladder cancer cells and the polarization of M2 macrophages

**Figure 6:**
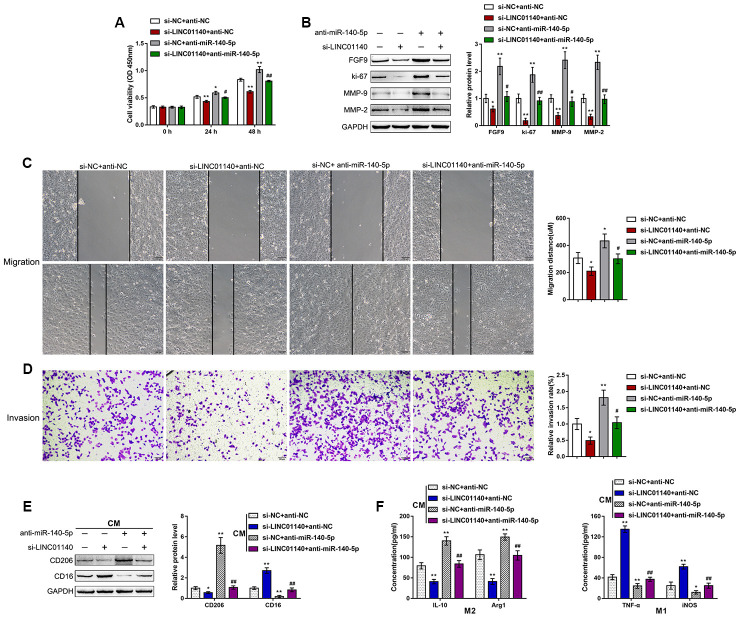
****Dynamic effects of LINC01140 and miR-140-5p on bladder cancer cell aggressiveness and macrophage M2 polarization T24 cells were cotransfected with si-LINC01140 and anti-miR-140-5p and examined for (**A**) the cell viability by MTT assay; (**B**) protein levels of FGF9, ki-67, MMP-2, and MMP-9 by immunoblotting; (**C**) migration capacity by wound healing assay; and (**D**) invasive capacity by Transwell assay. (**E**, **F**) T24 cells were cotransfected with si-LINC01140 and anti-miR-140-5p and the culture medium (shown in the figures as conditioned medium (CM) si-NC + anti-NC, si-LINC01140 + anti-NC, si-NC + anti-miR-140-5p, and si-LINC01140 + anti-miR-140-5p) was collected for macrophage incubation. M0 macrophages were cultured in the above-described four kinds of CMs and polarized to M1 or M2, respectively, and examined for (**E**) the protein levels of CD206 and CD16 by immunoblotting. (**F**) The concentrations of IL-10, Arg1, iNOS, and TNF-α in the macrophage culture medium was determined by ELISA. *P<0.05, **P<0.01, compared to control group; #*P*<0.05, ##*P*<0.01, compared to si-NC + anti-miR-140-5p group. One way ANOVA test was used for statistical analysis.

First, we confirmed the binding of miR-140-5p and LINC01140 to the FGF9 3'UTR. Next, we continued to investigate the dynamic effects of LINC01140 and miR-140-5p on FGF9 protein levels and bladder cancer cells. We cotransfected T24 cells with si-LINC01140 and anti-miR-140-5p and then determined the related indexes. LINC01140 knockdown significantly suppressed, whereas the inhibition of miR-140-5p enhanced T24 cell viability (Figure 6A), migration capacity (Figure 6C), and invasive capacity (Figure 6D); the roles of LINC01140 knockdown showed to be significantly reversed via the inhibition of miR-140-5p. Similarly, LINC01140 knockdown significantly suppressed FGF9, ki-67, MMP-2, and MMP-9 protein levels, while the inhibition of miR-140-5p enhanced FGF9, ki-67, MMP-2, and MMP-9 protein levels; the effects of LINC01140 knockdown were significantly reversed by the inhibition of miR-140-5p (Figure 6B). Regarding macrophage M2 polarization, T24 cells were cotransfected with si-LINC01140 and anti-miR-140-5p and the culture medium (shown in the figures as conditioned medium (CM) si-NC + anti-NC, si-LINC01140 + anti-NC, si-NC + anti-miR-140-5p, and si-LINC01140 + anti-miR-140-5p) was collected for macrophage incubation. M0 macrophages were cultured in the above-described four kinds of CMs and polarized to M1 or M2, accordingly. Under M2 polarization conditions, culture in si-LINC01140 + anti-NC CM significantly decreased the expression of the M2 marker CD206 (Figure 6E) and the concentration of IL-10, and Arg1 in the culture media (Figure 6F). In contrast, culture in si-NC + anti-miR-140-5p CM increased M2 marker levels, including CD206 (Figure 6E), IL-10, and Arg1 (Figure 6F). Under M1 polarization conditions, culture in si-LINC01140 + anti-NC CM decreased the M1 markers TNF-α and iNOS (Figure 6F). Notably, under si-LINC01140 + anti-miR-140-5p CM, the effects of LINC01140 knockdown were significantly reversed by the inhibition of miR-140-5p (Figure 6E–6G), indicating that LINC01140 targets miR-140-5p to counteract miR-140-5p-mediated suppression of FGF9, therefore promoting bladder cancer cell aggressiveness and macrophage M2 polarization.

## DISCUSSION

FGF9 (fibroblast growth factor 9) belongs to the human FGF family and plays a critical role in embryo development, tissue repair, tumorigenesis and many other biological functions [33–35]. FGF9 is identified as an important epithelial-to-mesenchymal transition signal required for embryo development [36], furthermore, it has been reported to be expressed in various tumors, such as colon cancer, gastric carcinoma and lung carcinoma [37–39]. FGF9 has carcinogenic activities, which support a function for its increase in the pathogenesis of several cancers. Transfecting human fibroblast growth factor 9 (FGF-9) cDNA into mouse BALB/c 3T3 clone A31 cells could lead to morphological transformation of the cells, formation of foci, soft agar growth and tumor formation within nude mice [40]. In bladder cancer, online data analysis indicated that the expression of FGF9 was markedly increased in MIBC tissues compared within NMIBC tissues, suggesting that FGF9 upregulation might be related to the aggressiveness of bladder carcinoma cells. As previously mentioned, high M2-like polarized macrophage infiltration correlated with worse clinical outcomes in bladder carcinoma patients [19]. M2 macrophages are the main type of bladder cancer-related macrophages and can accelerate bladder cancer progression [18, 19]. Notably, herein, macrophage M2 polarization was more frequently observed in MIBC tissues. Reportedly, FGF9 can enhance M2 macrophage polarization in the infarcted diabetic heart [17]; thus, it is reasonable to speculate that FGF9 might modulate macrophage M2 polarization, therefore affecting bladder cancer cell aggressiveness. As expected, FGF9 knockdown in T24 bladder carcinoma cells remarkably suppressed the viability, migratory capacity and invasive capacity of cells. More importantly, after incubation with conditioned medium derived from FGF9 knockdown T24 cells (si-FGF9-CM), the M2 polarization of macrophages was blocked, suggesting that abnormal FGF9 upregulation in bladder cancer might promote macrophage M2 polarization to accelerate cancer development.

LncRNAs are essential for tumorigenesis [41]. By modulating gene expression via transcription, posttranscriptional processing, chromatin modification, protein function modulation and many other mechanisms, lncRNAs contribute to various tumor initiation processes [42, 43]. Bladder cancer also shows changes in the expression levels of multiple lncRNAs. Various *in vitro* and *in vivo* investigations have evaluated the effects of knocking down RNAs in cancer cell phenotypes and *in vivo* tumor growth [44]. Interestingly, lncRNAs, including MM2P [31], MALAT1 [30], GNAS-AS1 [45], and SNHG12 [46], have been reported to modulate macrophage M1/2 polarization [47, 48]. According to online data, the expression of lncRNA LINC01140 was obviously increased in MIBC and had a positive correlation with the expression of FGF9, suggesting an underlying effect on bladder carcinoma. Song et al. [49] reported that a higher level of LINC01140 was linked to a decreased survival of gastric carcinoma patients. Moreover, it has been revealed by gene set enrichment analysis revealed that high risk scores had a positive correlation with some molecular pathways of cancer metastasis. In the present study, LINC01140 knockdown in T24 cells significantly inhibited the viability, migratory capacity, and invasive capacity of cells. Similarly, culture with si-LINC01140-CM inhibited macrophage M2 polarization, indicating that LINC01140 might cooperate with FGF9 to modulate macrophage M2 polarization in bladder cancer, therefore affecting cancer cell aggressiveness.

The delineation of the mechanism by which lncRNAs regulate gene expression remains a major challenge. As we have mentioned, lncRNAs can regulate gene expression through transcription, posttranscriptional processing, chromatin modification, protein function modulation and many other mechanisms. However, there is increasing evidence that lncRNAs can competitively target miRNAs via their MREs (miRNA response elements) to act as ceRNAs (competing endogenous RNAs), thus regulating the expression of miRNA downstream target RNAs [32]. Since LINC01140 and FGF9 expression was positively correlated in bladder cancer tissues, the present study further searched for miRNAs that might simultaneously target both LINC01140 and FGF9. Of the two candidate miRNAs, miR-140-5p and miR-580-3p, the expression of miR-140-5p was found to be dramatically downregulated in T24 cells compared with normal cells. Moreover, experimental results confirmed the online tool predictions that LINC01140 targets miR-140-5p, while miR-140-5p targets FGF9. Thus, LINC01140 might relieve miR-140-5p-induced inhibition of FGF9 by acting as a ceRNA, therefore affecting macrophage M2 polarization in bladder cancer.

miR-140-5p is known for its tumor suppressive effect on a variety of cancers, such as hepatocellular carcinoma [50], ovarian carcinoma [51], colorectal cancer [52, 53], and hypopharyngeal squamous cell carcinoma [54]. Interestingly, it has been shown that miR-140-5p targets FGF9 to inhibit hepatocellular carcinoma growth and metastasis [50]. Knockdown FGF9 via siRNA (small interfering RNA) resulted in a similar phenotype as ectopic miR-140-5p expression, whereas FGF9 overexpression reversed the effects of miR-140-5p in hepatocellular carcinoma growth and metastasis [50]. Accordingly, miR-140-5p inhibition in T24 cells significantly promoted the viability, migratory capacity and invasive capacity of cancer cells. In contrast to si-LINC01140-CM or si-FGF9-CM, anti-miR-140-5p-CM significantly promoted macrophage M2 polarization. Overall, the inhibition of miR-140-5p reversed the effects of LINC01140 knockdown on FGF9 expression, bladder cancer phenotype, and macrophage M2 polarization, indicating that miR-140-5p mediates the crosstalk between LINC01140 and FGF9, thus modulating macrophage M2 polarization to affect bladder cancer cell aggressiveness.

**Figure 7:**
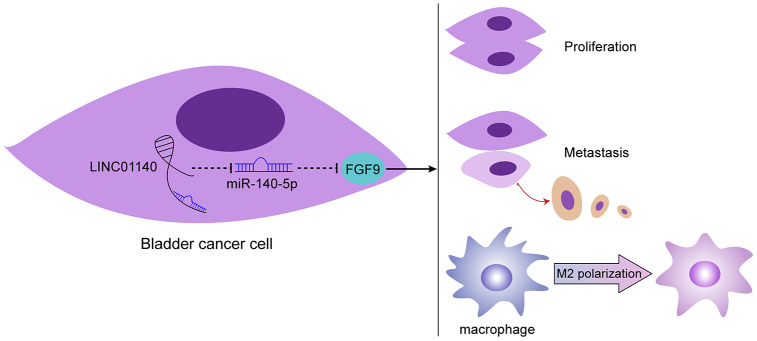
**Schematic diagram of the proposed mechanism.** LINC01140/miR-140-5p/FGF9 axis modulates bladder cancer phenotype and promotes macrophage M2 polarization through tumor microenvironment.

In conclusion, LINC01140, miR-140-5p, and FGF9 form a lncRNA-miRNA-mRNA axis that modulates the bladder cancer phenotype, affects macrophage M2 polarization through the tumor microenvironment, and in turn affects bladder cancer cell aggressiveness (Figure 7). However, the application of this axis needs further *in vivo* and clinical investigation.

## MATERIALS AND METHODS

### Clinical sampling

Muscular invasive bladder cancer (MIBC) tissue samples from 12 cases and non-muscular invasive bladder cancer (NMIBC) tissue samples from 12 cases were obtained from patients clinically and histologically diagnosed at The Second Xiangya Hospital with the approval of the institutional Ethics Committee. Written informed consent was obtained from each patient enrolled. The clinic character was shown in Supplementary Table 1.

### Cell lines and cell transfection

Human bladder epithelial SV40 immortalized cell line, SV-HUC-1 (ATCC® CRL-9520), was obtained from ATCC (Manassas, VA, USA) and cultured in F-12K Medium (Catalog No. 30-2004, ATCC) supplemented with 10% FBS. A bladder cancer cell line, T24 (ATCC® HTB-4), was obtained from ATCC and cultured in McCoy's 5a Medium Modified (Catalog No. 30-2007, ATCC) supplemented with 10% FBS. All cells were cultured at 37° C in 5% CO_2_.

The expression of miR-140-5p was modulated by transfection of miR-140-5p or anti-miR-140-5p (GenePharma, Shanghai, China). FGF9 or LINC01140 was knocked down by transfection of si-FGF9 or si-LINC01140 (GenePharma). All transfections were performed using the transfection agent Lipofectamine 3000 (Invitrogen). The relative sequences are shown in Supplementary Table 2.

### Bioinformatic analysis

To identify genes related to MIBC, we downloaded a microarray expression profile dataset, GSE77952, from Gene Expression Omnibus (GEO). This dataset reported the differentially expressed genes, including protein-coding RNAs and noncoding RNAs, between 14 cases of MIBC and 16 cases of NMIBC tissues. In this dataset, a total of 45 cytokines were deregulated in MIBC; after literature review, FGF9 was selected for further experiments.

### Survival analysis

To analyze the correlation of FGF9 or LINC01140 expression with the overall survival in patients with bladder cancer, we used the American National Cancer Institute's genomic data sharing port database (GDC, Genomic Data Commons; https://portal.gdc.cancer.gov/). We selected TCGA-BLCA from the Project menu on the repository page, downloaded the transcriptome data (transcriptome profiling) from the Files menu, and then selected RNA-seq (HTseq-Counts) Public data; the total number of samples was 434. The R language tool was used to read the sequencing data of each sample and integrate it into an expression matrix. The expression matrix was then homogenized with the DEseq2 and Edge R packages for a standardized expression matrix. Normalized gene expression data for FGF9 (ENSG00000102678) and LINC01140 (ENSG00000267272) were extracted from this matrix. The survival curve was obtained by the survival and survminer R packages. The raw survival data were analyzed using the R language package (RTCGA.clinical). The parameters used were patient survival (patient.vital_status), the time from follow-up to death (patient.ups. Follow_up.days_to_death), and the time the patient had survived at the last follow-up (patient.ups. Follow_up.days_to_last_followup). All the above parameters are expressed in days. When dealing with survival data, cases with a survival time of less than 30 days or missing values were removed because a survival time of less than 30 days might be represented death due to rapidly deteriorating health post surgery, and may not be directly related to tumor gene expression. Finally, 286 patients were analyzed. When grouping the cases by the median value of gene expression, the minimum value of the ratio of high and low expression groups was 30/70, that is, the two groups of samples had to distributed between 30% and 70% (minprop = 0.3) to ensure that there was no large deviation between the two sets of samples. Univariate Cox regression analysis was used to analyze the overall survival.

### PCR-based analysis

Total RNA was extracted from target tissues or cells using TRIzol reagent (Cat:15596018, Invitrogen). After that, the expression of lncRNA, miRNA, and mRNA was determined by real-time PCR using a SYBR Green qPCR assay (Cat.DRR820A, Takara, Dalian, China) following the methods described before [55]. The expression of RNU6B or GAPDH was used as an endogenous control for miRNA or mRNA expression calculations. The data processing and relative fold-change calculation were generated using the 2^-ΔΔCT^ method. The primer sequences are listed in Supplementary Table 2.

### Immunoblotting

To monitor the changes in the protein levels of FGF9, ki-67, MMP-2, and MMP-9, we performed immunoblotting following the methods described before [55] with antibodies against CD206, CD16, FGF9, ki-67, MMP-2, and MMP-9 (ab64693, ab46679, ab71395, ab15580, and ab38898, Abcam, Cambridge, UK) followed by another incubation with the appropriate HRP-conjugated secondary antibodies. GAPDH (ab16891, Abcam) was used as an endogenous control. Signals were visualized using enhanced chemiluminescent (ECL) substrates (WBKlS0100, Millipore, MA, USA) and were normalized to the GAPDH signal.

### Hematoxylin and eosin (H&E) staining

MIBC and NMIBC tissues were fixed in 4% paraformaldehyde for 24 h. Then, the tissue blocks were dehydrated, embedded in paraffin, and cut into 4-μm-thick slices. After dewaxing, the slices were stained with H&E using standard procedures [56, 57].

### Immunofluorescence (IF) staining for bladder cancer tissues

For the detection of CD163 in bladder cancer tissues, tissue samples were fixed in 4% formaldehyde and embedded in paraffin. Tissues were blocked with 100 mM ammonium chloride buffer for 10 min to minimize autofluorescence. Nonspecific binding sites were blocked with PBS containing 10% horse serum followed by an incubation with the primary antibody specific to CD163 (ab87099, Abcam) overnight at 4° C, diluted in blocking solution. Cy3-conjugated goat anti-rabbit secondary antibody (A0516, Beyotime, China) was incubated at room temperature for 1 h. DAPI (C1002, Beyotime, China) was used to stain the nucleus before capturing images. The images were acquired using a fluorescence microscope (Nikon, Japan). The red fluorescence indicates CD163 expression, and the blue fluorescence indicates the nucleus.

### Monocyte isolation and differentiation into macrophages

Human peripheral blood mononuclear cells (PBMCs) were obtained from the venous blood of healthy volunteers and separated through Ficoll density gradient centrifugation. Next, monocytes were isolated from PBMCs using anti-CD14 magnetic beads (Life Technologies, USA) following the manufacturer's instructions. The nonadherent cells were removed, and purified monocytes were cultured in RPMI 1640 medium supplemented with 10% FBS for one week. Then, 50 ng/ml macrophage-colony stimulating factor (M-CSF; R&D Systems, USA) was used for seven days to obtain M0 macrophages. To stimulate M0 macrophages to differentiate into M1 or M2 macrophages, the study stimulated M0 macrophages with 100 ng/ml LPS (Sigma-Aldrich) + 20 ng/ml IFN-γ (eBioscience) for M1 polarization induction or 20 ng/ml IL-4 (eBioscience) for M2 polarization for 48 h following the methods described previously [58–60].

For macrophage surface markers determination by flow cytometry, cells were stained with an anti-CD11b (ab24874, Abcam), anti-CD206 (ab223960, Abcam) or anti-CD16 (ab234209, Abcam) fluorophore-conjugated antibody. Samples were then quantified using flow cytometry analysis of macrophage cell surface markers in a FACSCalibur flow cytometer (BD Biosciences). The isotype controls were shown in Supplementary Figure 1. For macrophage surface makers determination by IF, cells were seeded onto coverslips overnight, fixed with 4% paraformaldehyde PBS and incubated with anti-CD11b (ab8878, Abcam), anti-CD206 (ab64693, Abcam) or CD16 (ab46679) at 4° C overnight. FITC-conjugated or TRITC-conjugated secondary antibody (Beyotime, China) was incubated at room temperature for 1 h. The nuclei were stained with DAPI (KeyGen Biotech). Images were taken with a fluorescence microscope.

### Cell viability evaluated by MTT assay

By following the methods described previously [61], cell viability was detected. After transfection or treatment, MTT (20 μl at a density of 5 mg/ml; Sigma-Aldrich) was added, and another 4-h incubation was conducted. At the end of the incubation, DMSO (200 μl) was added to dissolve the formazan. Next, OD values were measured at 490 nm and the relative cell viability was calculated by defining the nontreated cell viability (control) as 100%.

### Cell migratory capacity evaluated by wound healing assay

Cells were seeded in 6-well plates at 5 × 10^5^ cells/ml, until the cells formed a monolayer, and then the cell wound healing assay was performed. The scratch area was measured under a microscope (Olympus, Japan) at 0 h and 48 h. The relative distance of cell migration to the scratch area was also measured under the microscope and analyzed by ImageJ software (NIH, USA).

### Cell invasive capacity evaluated by Transwell assay

Cells (5 × 10^5^) were plated on the top side of polycarbonate Transwell filters coated with Matrigel. For Transwell invasion assays, cells were suspended in medium without serum and medium with serum was added to the bottom chamber. The cells were incubated at 37° C for 48 h. The noninvasive cells in the top chambers were removed with cotton swabs. The invasive cells on the lower membrane surface were fixed in 100% methanol for 10 min, air-dried, stained with crystal violet solution (C0121, Beyotime), and then counted under a microscope.

### ELISA

Culture supernatants were harvested and centrifuged to remove cellular debris, and aliquots were stored at –80° C until assayed. The concentrations of IL-10, Arg1, iNOS, and TNF-α were determined using corresponding human ELISA kits (IL-10: D1000B, R&D Systems; Arg1: P05089, Cusabio, Houston, TX, USA; iNOS: P35228, Cusabio; TNF-α: DTA00D, R&D Systems) following the methods described previously [62].

### Luciferase reporter assay

To validate the binding between miR-140-5p and LINC01140 or the 3'UTR of FGF9, the wild-type or mutated LINC01140 or the 3'UTR of FGF9 was cloned downstream of the Renilla gene in the psiCHECK2 vector (Promega, Madison, WI, USA), and the resulting constructs were named wt-LINC01140, wt-FGF9 3'UTR, mut-LINC01140 or mut-FGF9 3'UTR. Next, 293T cells were co-transfected with the two types of luciferase reporter vectors and miR-140-5p mimics/miR-140-5p inhibitor and examined for luciferase activity using the Dual-Luciferase Reporter Assay System (Promega).

### Data processing and statistical analysis

The data were analyzed with GraphPad software. The measurement data are expressed as the mean ± standard deviation (SD). Intergroup and intra-group data comparisons were performed with the ANOVA and Student’s *t*-tests. *P*<0.05 indicates a statistically significant difference.

## Supplementary Material

Supplementary Figures

Supplementary Tables
