# Supplementary material for: The long noncoding RNA LINC01140/miR-140-5p/FGF9 axis modulates bladder cancer cell aggressiveness and macrophage M2 polarization
Source: Aging (Albany NY). 2020 Nov 21;12(24):25845–64. doi: 10.18632/aging.202147 (PMC7803526; doi:10.18632/aging.202147)
Supplement: Supplementary Figures [file aging-12-202147-s001.pdf]

SUPPLEMENTARY FIGURES

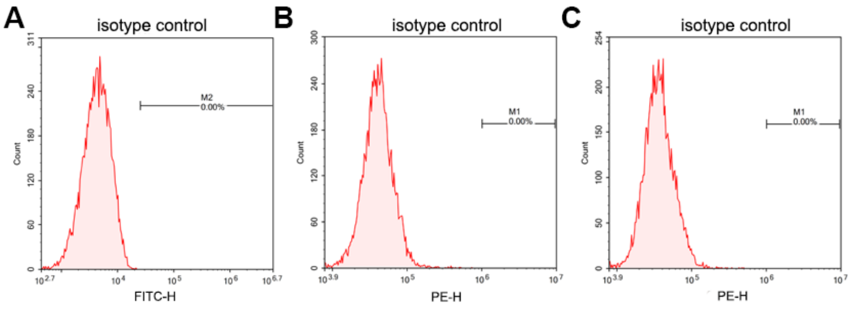

Supplementary Figure 1. Isotype control for flow cytometry. (**A**) Rat IgG isotype control-FITC (ab136125); (**B**) Rabbit IgG isotype control -PE (ab209478). (**C**) Mouse IgG isotype control-PE (ab154457).

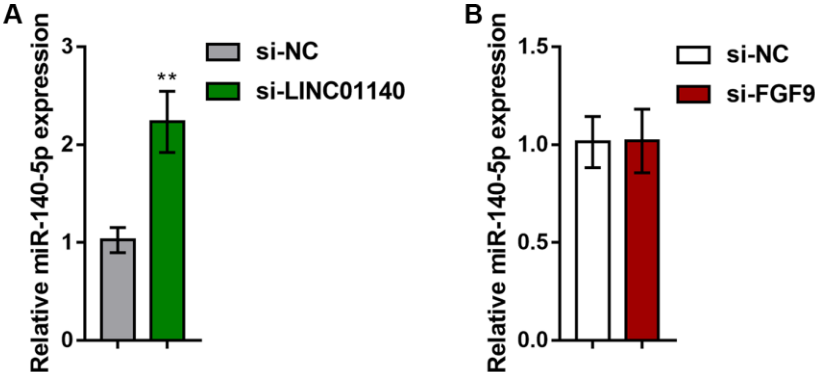

Supplementary Figure 2. Relevance of miR-140-5p to LINC01140 and FGF9. (**A**)The levels of miR-140-5p in response to LINC01140 knockdown; (**B**) The levels of miR-140-5p in response to FGF9 knockdown in bladder cancer cells.
