# Supplementary material for: The long noncoding RNA LINC01140/miR-140-5p/FGF9 axis modulates bladder cancer cell aggressiveness and macrophage M2 polarization
Source: Aging (Albany NY). 2020 Nov 21;12(24):25845–64. doi: 10.18632/aging.202147 (PMC7803526; doi:10.18632/aging.202147)
Supplement: Supplementary Tables [file aging-12-202147-s002.pdf]

SUPPLEMENTARY TABLES

Supplementary Table 1. Clinical characteristics of patients.

No.	Gender	Age	Single/Multiple	TNM stage	Diagnosis
1	M	21	multiple	T1N0M0	NMIBC
2	M	63	multiple	T1N0M0	NMIBC
3	F	58	single	T1N0M0	NMIBC
4	F	61	multiple	T1N0M0	NMIBC
5	F	53	single	T1N0M0	NMIBC
6	M	62	multiple	T1N0M0	NMIBC
7	F	56	single	T1N0M0	NMIBC
8	F	53	single	T1N0M0	NMIBC
9	M	64	multiple	T1N0M0	NMIBC
10	F	66	single	T1N0M0	NMIBC
11	F	51	single	T1N0M0	NMIBC
12	M	54	single	T1N0M0	NMIBC
13	M	64	multiple	T3N1M0	MIBC
14	F	75	single	T3N0M0	MIBC
15	M	56	multiple	T2N1M0	MIBC
16	M	65	single	T2N0M0	MIBC
17	F	67	single	T3N0M0	MIBC
18	M	57	multiple	T2N1M0	MIBC
19	M	71	single	T3N0M0	MIBC
20	M	68	single	T2N0M0	MIBC
21	M	50	multiple	T3N0M0	MIBC
22	M	59	single	T2N0M0	MIBC
23	M	39	single	T2N0M0	MIBC
24	M	68	multiple	T2N1M0	MIBC

Supplementary Table 2. The primers sequence.

name	Forward 5'-3'	Reverse 5'-3'
MiR-140-5p mimics	CAGUGGUUUUACCCUAUGGUAG	ACCATAGGGTAAAACCACTGUU
miR-140-5p inhibitor	CUACCAUUGGGUAAAACCACUG	
Mimics NC	UUC UCC GAA CGU GUC ACG UTT	ACG UGA CAC GUU CGG AGA ATT
Inhibitor NC	CAGUACUUUUGUGUAGUACAA	
QPCR-FGF9	ATGGCTCCCTTAGGTGAAGTT	CCCAGGTGGTCACTTAACAAAAC
QPCR-LINC01140	CAGGAGAGACACAGACTTGGGG	CACCCCGAGCGATGGAGTA
QPCR-miR-140-5P	RT:GTCGTATCCAGTGCGTGTCGTGGAGTCGGC<br>AATTGCACTGGATACGACCTACCA<br>GCCAGTGGTTTTACCCTA	CAGTGCGTGTCGTGGA
QPCR-GAPDH	ACAGCCTCAAGATCATCAGC	GGTCATGAGTCCTTCCACGAT
QPCR-U6	CTCGCTTCGGCAGCACA	AACGCTTCACGAATTTGCGT
Si-NC	UUC UCC GAA CGU GUC ACG UTT	ACG UGA CAC GUU CGG AGA ATT
Si-FGF9	GCGAUACUAUGUUGCAUUATT	UAAUGCAACAUAGUAUCGCTT
Si-LINC01140	CCAACUAUUUCAAAUGCAATT	UUGCAUUUGAAAUAGUUGGTT
